# Home Cardiotocography in High-Risk Pregnancies: A Retrospective Study on Feasibility and Patient Well-Being

**DOI:** 10.1089/tmr.2024.0062

**Published:** 2025-01-14

**Authors:** Małgorzata Olesiak-Andryszczak, Jakub Pytlos, Anna Suchecka-Marut, Małgorzata Świdzińska, Marzena Mazurek

**Affiliations:** ^1^ Regional Specialist Hospital in Wrocław, Research and Development Center, Wrocław, Poland.; ^2^ Sonokard Medical Center, Wrocław, Poland.; ^3^ Wolski Hospital, Warsaw, Poland.; ^4^ MyClinic, Out-patient Private Clinic, Warsaw, Poland.

**Keywords:** tele-CTG, home-CTG, telemedicine, cardiotocography, high-risk pregnancy, patient-centered care, pregnancy complications

## Abstract

**Background:**

Around 15% of pregnancies develop complications requiring detailed monitoring. With technological advancements, home cardiotocography has emerged as a potential alternative to the conventional approach, aiming to alleviate the stress and logistical challenges associated with in-clinic care. The objective of this study was to evaluate the capability of a portable device to collect data for accurate interpretation of the examination and to evaluate patient satisfaction, along with physical and emotional comfort during the monitoring process.

**Methods:**

This retrospective study included 32 women with high-risk pregnancies, who independently performed 581 examinations utilizing the portable cardiotocography device Pregnabit Pro (Nestmedic, Poland). Moreover, participants were asked to provide feedback on their experiences through a comprehensive survey.

**Results:**

In total, 95.7% of examinations successfully captured all necessary diagnostic data. Patients reported a high satisfaction rate, recognizing the potential of the home-based approach to improve their overall pregnancy experience. A preference for home-based testing over in-clinic visits was consistently noted among patients.

**Discussion:**

This study shows evidence that home cardiotocography is a feasible and patient-preferred method for monitoring high-risk pregnancies. Our findings underline the importance of technological integration in prenatal care, advocating for a shift toward more patient-centered, accessible, and potentially cost-effective health care solutions.

## Introduction

While most pregnancies proceed without complications, approximately 15% of all pregnant women develop conditions that affect the health of the mother or her fetus.^1^ A high-risk pregnancy is defined as presenting maternal or fetal factors that have the potential to adversely impact the outcome of the pregnancy.^2^ Women diagnosed with pregnancy complications are recommended to undergo frequent check-up visits and intensive monitoring of both maternal and fetal parameters.^3^ A thorough assessment of these parameters is achieved with tests such as cardiotocography (CTG), which often requires prolonged hospital stays or regular visits to the outpatient clinic.

CTG is a commonly used diagnostic procedure for monitoring the well-being of the fetus. It is painless, noninvasive, and entails no risk of adverse events for both the patient and the fetus. The examination is comprised of two components: fetal heart rate monitoring with a Doppler ultrasound transducer (cardio) and the external monitoring of uterine contractions using a tocodynamometer (toco). The examination is often done simultaneously with monitoring of the maternal heart rate to ensure the distinction between heart rates.^4^ The classical CTG recorder is a stationary system, thus formerly the check-up CTG examinations have been conducted exclusively in specialized clinics or perinatal departments.

With advancements in the portability of medical devices, hospital admissions are no longer required for conducting a CTG test. Hospitalization is often undesirable, as patients with high-risk pregnancies report greater anxiety, lower self-esteem, and impaired family functioning while hospitalized.^5,6^ This is likely caused by factors such as a stay in an unfamiliar place, separation from family and the need to arrange childcare, which exacerbate stress.^2^ Home pregnancy care not only diminishes the necessity for frequent hospital visits, improving accessibility of health care and reducing clinic expenses, but also increases compliance with scheduled appointments and fosters greater patient engagement.^7^

The aim of this retrospective study is to assess whether examination with a portable CTG device enables the collection of data necessary for its accurate interpretation, presenting a viable alternative to conventional testing during hospital admissions for high-risk pregnancies. Furthermore, our objective was to ascertain the acceptability of home CTG among patients and its potential to enhance the physical and emotional comfort of patients undergoing the monitoring process.

## Methods

### Study design and participants

**FIG. 1.**
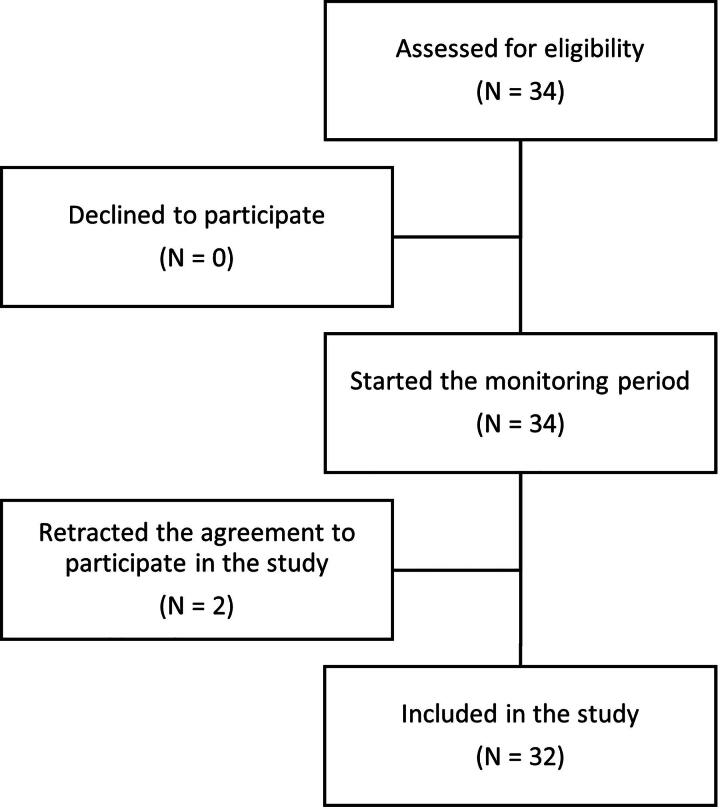
Participant flow diagram.

This retrospective study included 32 women with high-risk pregnancies, who individually conducted 581 tests with mobile CTG devices, between January and June 2023. Eligible were women aged 18 years and older with a singleton pregnancy (>32 + 0 weeks gestational age) who required maternal or fetal surveillance due to one (or multiple) of the following reasons: pregnancies with fetal growth restriction, chronic or pregnancy-induced hypertension, pregestational diabetes mellitus, and gestational diabetes mellitus. In addition, only patients capable of autonomously conducting a home CTG test following instructions provided by a midwife were included in the study. Exclusion criteria were: patient requiring hospitalization or life support, contraindications for CTG testing, allergies to ultrasound gel or latex, presence of an active medical implant, and acute or chronic skin alterations or wounds at the site of contact between the CTG device and body of the patient. The participant flowchart is shown in Figure 1.

All participants were comprehensively informed about the study and provided written informed consent to participate.

### Procedures and devices

**FIG. 2.**
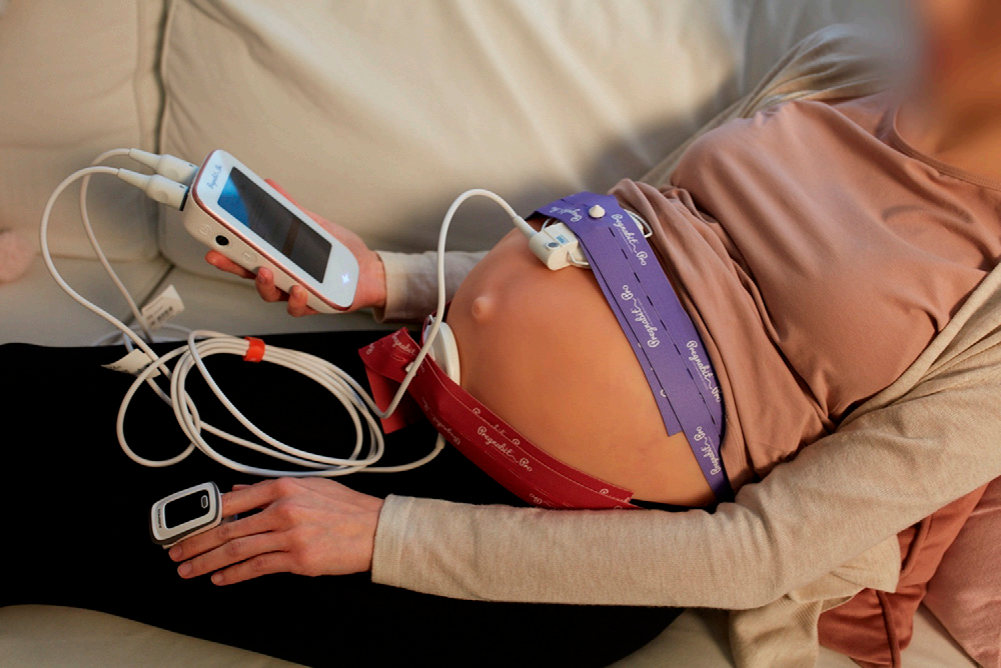
Exemplary patient during an examination with the device. The photo was provided by the Nestmedic S.A.

The CTG examinations were conducted using the Pregnabit Pro device (Nestmedic, Poland), integrated with the Pregnabit Cloud telemedicine system (Fig. 2). The device utilizes two separate belts, one attached to a Doppler ultrasound transducer and the other to a tocodynamometer transducer. In addition, the device uses an integrated pulse oximeter to capture the maternal heart rate. Patients are responsible for independently positioning all three transducers.

As a part of routine prenatal care in our clinic, patients were encouraged to enroll in the home CTG monitoring program. Those who expressed interest received a QR code from their attending gynecologist, directing them to the Pregnabit Pro website, where they completed a form providing their personal information. Medical personnel from the CTG monitoring center then initiated contact with the participants, established their patient accounts, and provided detailed instructions on performing home CTG tests. Subsequently, a courier delivered the Pregnabit Pro devices to the residences of the patients. In addition, patients received an enclosed written user manual and an instructional video recorded on the main screen of the device, demonstrating the correct preparation and execution of the examination. Access to the device, system, and medical staff expertise was provided to patients free of charge.

**FIG. 3.**
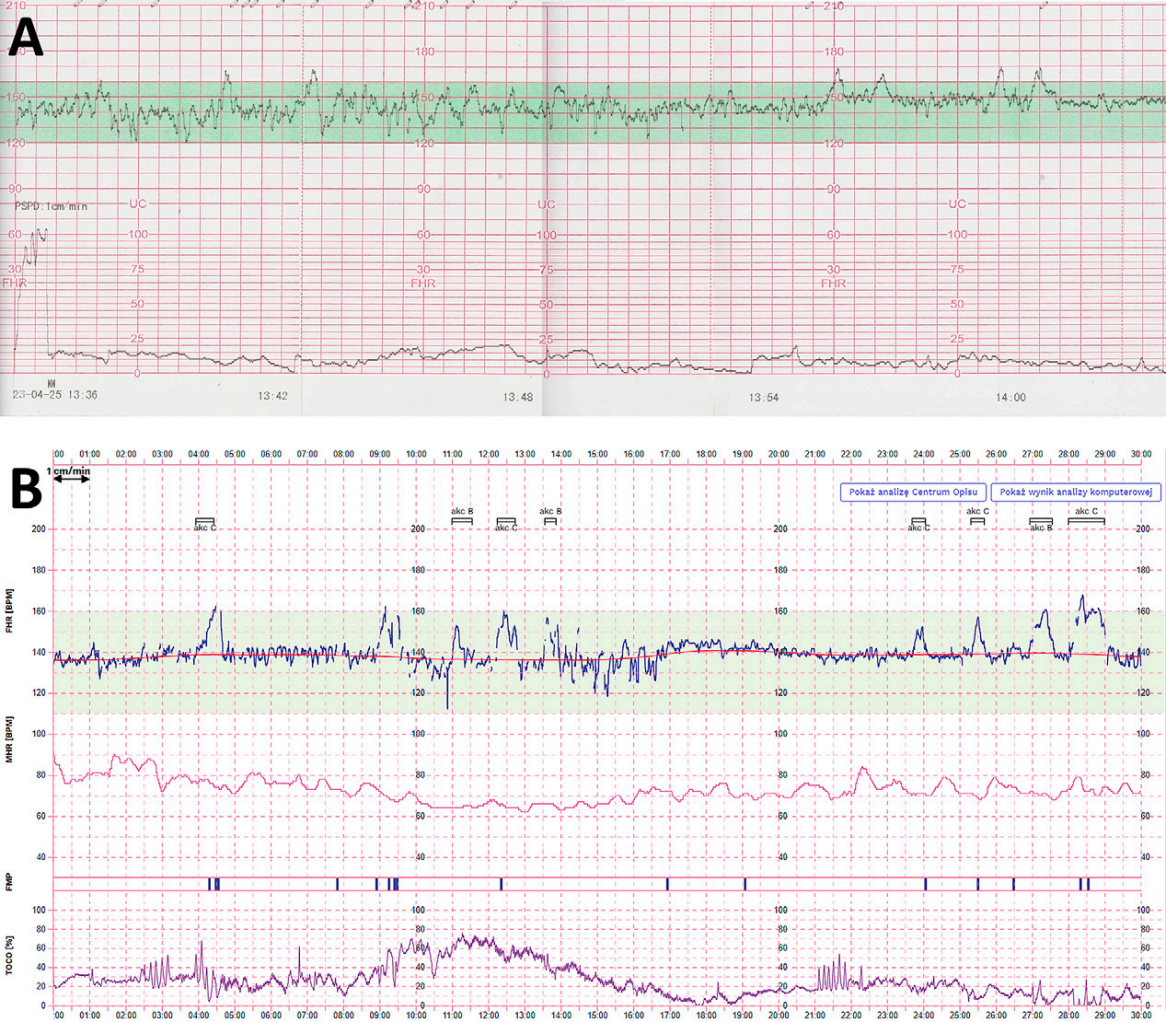
A comparison between a conventional CTG recording **(A)** versus a CTG recorded using the Pregnabit Pro device **(B)**. Examinations were conducted on the same participant. CTG, cardiotocography.

Patients used the device throughout the entire period from enrollment until labor. They were instructed to perform the examinations daily, but not required to adhere to a fixed time schedule. The CTG examinations were conducted in sessions lasting 30 min. In the event of signal loss from any component, the device had the capability to independently prolong the session duration or stop the examination entirely and request a repeat examination. Within the CTG recordings undergoing analysis, a 20-min interval was designated for assessment purposes. The personal data of the patients were anonymized and the device automatically transmitted the complete examination record through the integrated system for evaluation. The test results were independently analyzed by two qualified midwives and a specialist gynecologist in accordance with National Institute for Health and Care Excellence (NICE) and The International Federation of Gynecology and Obstetrics (FIGO) recommendations defining a good assessment of CTG recording.^4,8^ Following each examination, the patients were issued an SMS notification containing the results of the examination and further guidance. In case of an inaccurate recording or the necessity for a repetition, the medical staff contacted the patient via telephone. Patients were provided with the opportunity to reach out to their attending gynecologist or the monitoring center staff anytime for any queries or concerns. To ensure the safety of all participants, during weekly follow-up visits at the clinic, the patients underwent additional CTG examinations using stationary equipment, which were evaluated by a gynecologist (Fig. 3). The gynecologist overseeing the pregnant women had access to all examinations conducted by the patients using the Pregnabit Pro device and based on collected data, made decisions regarding further recommendations for the pregnant woman until the time of hospitalization.

Upon the conclusion of the monitoring period, examined patients who met the eligibility criteria were invited to participate in a retrospective study utilizing the data collected. Comprehensive information about the study was provided, and those who expressed interest provided written informed consent to participate. Subsequently, patients enrolled in the study were asked to provide feedback about the monitoring process through a comprehensive survey detailing their individual experiences of home CTG examinations and hospitalization (Supplementary Data S1).

### Outcomes

The primary outcome was the feasibility of CTG testing with a portable device. Achieving it required a detailed analysis to determine whether the signals captured by the device included the necessary components for a precise CTG interpretation. The evaluation was guided by a protocol (list of inquiries) defining the successive stages of fetal well-being assessment: detection of fetal heart rate, detection of maternal heart rate, registration of fetal movements, identification of uterine contractile activity, observation of oscillations, assessment of the range of oscillation variability, and determination of values of maternal and fetal heart rates. The examination was considered feasible on the condition that the panel of three specialists unanimously confirmed that the recording included all signal components necessary for interpretation.

A separate outcome was patient wellbeing and acceptance of the home CTG method, assessed by the conducted survey. The survey questions were formulated with the intention of investigating the perception of pregnant women regarding the novel method of pregnancy monitoring. These questions included assessing the ease of independent test performance at home, evaluating the need for patient contact with medical personnel at the monitoring center, comparing the levels of physical comfort between home CTG examinations and tests performed at the medical center, as well as appraising the potential enhancement of the sense of security and psychological comfort attributed to daily home CTG testing.

### Data analysis

Each step of data analysis was performed by JP. Responses to the open-ended questions included in the survey were organized into transcripts using open coding categories, assigned to text fragments. Article was prepared using the SQUIRE checklist (Supplementary Data S2).^9^

### Ethics

The trial protocol was approved by the Bioethics Committee of the Regional Chamber of Physicians and Dentists in Wrocław (trial reference number 4/BNR/2023).

## Results

The 32 patients who agreed to participate in the study independently conducted a total of 581 home CTG examinations. The mean age of the patients was 32 years, with the range spanning from 23 to 42 years of age. Among the participants, 15 individuals (47%) were experiencing their first pregnancy, 10 (31%) were in their second, 2 in their third, 2 in their fourth, 2 in their fifth, and 1 in her sixth. The most common indications for participants to be enrolled were intrauterine growth restriction (16 [50%]), gestational diabetes (15 [47%]), and hypertension (9 [28%]). Notably, several patients exhibited multiple factors contributing to the high risk of their pregnancy. The median gestational age at which participants were enrolled in the study was 34 weeks and the median duration of inclusion in the study was 20.5 days (on average 23.6 days). In that time, patients conducted a median of 13.5 examinations (on average 18.2 examinations). Only one patient conducted fewer than one examination every 2 days on average. All 32 patients experienced successful deliveries.

Across 581 home CTG examinations, which were accepted by the device, a majority of 556 instances (95.7%) exhibited the complete set of required elements as per the protocol, recognized unanimously by all three specialists.

Fetal heart rate detection was successful in all 581 examinations, whereas maternal heart rate was detected in 580 examinations (99.8%). Fetal movements were registered in 579 examinations (99.7%). The uterine contractile activity was identified in 559 examinations (96.2%), indicating a slightly higher rate of error compared to other inquiries. Oscillations were observed consistently in all 581 tests, with the range in oscillation variability properly assessable across all conducted examinations. The values of maternal and fetal heart rates were determined in 580 examinations (99.8%) due to the one precedent where maternal heart rate was unsuccessfully detected. That CTG examination was the only instance where more than one criterion required for a successful test was not met. In 54 instances (9.3%), the device autonomously extended the duration of the examination to ensure an adequate length of time for recording all the necessary data.

Upon conclusion of the monitoring period, the participants filled in patient wellbeing questionnaires (Supplementary Data S1). The results of the patient surveys are shown in Table 1. In the first question, the women were asked about their ability to independently conduct the CTG examination following the phone/video training. Out of the respondents, 31 individuals (96.9%) reported no difficulty with performing the test, while one patient (3.1%) noted a necessity to seek guidance from the midwife at the monitoring center during the process. Subsequently, the participants were requested to evaluate, on a scale ranging from 1 to 10, their proficiency in detecting the fetal heartbeat using the device (10 meaning the full competence). The median rating reported was 9 (mean 8.5), with 10 women rating their proficiency with the maximum score of 10, followed by eight women rating it as 9, six as 8, five as 7, two as 6, and one patient selecting 5. Twenty-five (78.1%) participants felt that the CTG examinations proceeded without any disruptions. In the questionnaires, 25 participants (78.1%) expressed that according to their judgement, the CTG examinations were conducted without any disturbances. Patients that reported otherwise cited reasons such as frequent signal loss, challenges in locating the fetal heart rate due to numerous fetal movements, and technical problems. Fifteen participants (46.9%) reported instances where they had to repeat a CTG examination due to technical reasons. The reasons cited included multiple signal losses (reported by 12 patients, 37.5%), device discharges (2 patients, 6.3%), and device failure (1 patient, 3.1%). The necessity to contact the midwife at the monitoring center during the examination period was indicated by only two participants (6.3%). One of the patients needed to report a device failure, while the second patient encountered issues with logging into the telemedicine system.

**Table 1.** Results of the Survey for Patients Using the Pregnabit Pro Device

1. Were you able to independently perform the examination after the phone/video training?		
Yes, without any difficulty. I conducted the examination independently	31	96.88%
I needed additional help:		
Consultation with a Midwife	1	3.13%
Reading the user manual	0	0%
Watching the instructional video on the device	0	0%
I couldn’t perform the examination independently due to:		
The system being completely nonintuitive	0	0%
Unable to reach the midwife	0	0%
2. How do you rate the independent detection of the fetal heartbeat on a scale of 1 to 10?		
10	10	31.25%
9	8	25.00%
8	6	18.75%
7	5	15.63%
6	2	6.25%
5	1	3.13%
4 or less	0	0%
3. Did the CTG examinations proceed without disturbances?		
Without disturbances	25	78.13%
Examination interrupted:		
Multiple signal losses	5	15.63%
Technical Difficulties	2	6.25%
4. Have you ever had to repeat a CTG examination for technical reasons?		
No	17	53.13%
Yes, multiple signal losses	13	40.63%
Yes, Device discharge	2	6.25%
5. Did you feel the need to contact the Midwife during examinations?		
No	30	93.75%
Yes	2	6.25%
6. Which examination was more comfortable for you?		
Conducting the CTG examination independently at home	30	93.75%
CTG examination at a medical center	2	6.25%
7. Which statements below best describe your feelings during independent CTG examinations at home?		
Felt comfortable and safe	28	87.50%
Felt like nobody was monitoring the examinations	0	0%
Felt cared for by the midwife and doctor	23	71.88%
Felt abandoned	0	0%
Saved time compared to traveling to a medical facility	13	40.63%
Prefer stationary examinations	3	9.38%
Felt stressed during independent examinations	4	12.50%
Found preparation time-consuming and complicated	0	0%
Appreciated the opportunity for independent testing at home without hospital/clinic visits	10	31.25%
8. What difficulties did you encounter during the monitoring process?		
None	22	68.75%
Challenges with correctly positioning the Doppler ultrasound transducer	7	21.88%
Difficulty in accessing the telemedicine system	2	6.25%
Challenges in properly placing the tocodynamometer	1	3.13%
9. Is there anything that would improve your comfort during home CTG examinations?		
No	29	90.63%
Yes, improvements in the sensitivity of the Doppler ultrasound transducer	3	9.38%
10. How likely are you to recommend home CTG monitoring to peers or use it in your next pregnancy in the future?		
Very unlikely	0	0%
Rather unlikely	0	0%
Hard to say	0	0%
Rather probable	0	0%
Very probable	32	100%
11. Do you think any part of the procedure needs clarification?		
No	32	100%
Yes	0	0%
12. Do you find conducting independent CTG examinations easy or difficult?		
Easy	32	100%
Difficult	0	0%

The majority of patients (30 participants, 93.8%) expressed a preference for conducting independent CTG tests at home over CTG examinations in a medical center, with only two women (6.3%) opting for the latter. In a subsequent section of the questionnaire, the patients responded to a series of binary (yes or no) questions. The results were as follows: 28 women (87.5%) felt comfortable and secure throughout the examinations, none of the women (0%) felt that there was no appropriate monitoring by the medical personnel during the examinations, 23 women (71.9%) felt supported and cared for by the midwife and doctor, none of the women (0%) felt abandoned, 13 women (40.6%) mentioned that they saved time by avoiding the travel to a medical facility for the examination, only 3 women (9.4%) expressed a preference for stationary examinations, 4 women (12.5%) reported feeling stressed each time they conducted the examination independently, none of the women (0%) felt that preparation for the examination was time-consuming or complicated, and 10 women (31.3%) appreciated the opportunity for independent testing at home without the necessity of hospital or clinic visits. Patients were also queried about any challenges they faced during the monitoring process. A majority of 22 women (68.8%) responded that they experienced no difficulties. Seven women (21.9%) mentioned challenges with correctly positioning the Doppler ultrasound transducer to accurately measure the fetal heart rate. The remaining three women identified a difficulty in accessing the telemedicine system or challenges in properly placing the tocodynamometer. The patients were inquired about the existence of any factors that could enhance their comfort during home CTG examinations. Only three (9.4%) responded affirmatively, suggesting improvements in the sensitivity of the Doppler ultrasound transducer. Patients were asked to indicate how likely they would recommend home CTG monitoring to their peers or use it in their subsequent pregnancy. All 32 patients opted for the highest rating of “very likely” among the five available options. Furthermore, all patients affirmed that performing the CTG examinations independently was for them easy rather than difficult. None of the participants declared a necessity for further clarification regarding the method of conducting the test beyond the provided materials.

## Discussion

In this retrospective study, one of the few to investigate independent home CTG testing, we examined the feasibility and patient acceptance of home CTG examinations in high-risk pregnancies.

### Feasibility of CTG examinations with a portable device

Home management has been reported in earlier studies to be safe for most obstetric patients, including those with pregnancy-related complications.^10–13^ In addition to psychological benefits, home management has been shown to be a more cost-effective option, with estimated savings of 40–50% compared with hospital stays.^11^ Despite the advantages, transitioning from in-clinic to home-based management raises longstanding concerns among patients and physicians. Key concerns include the potential need for immediate medical intervention, risk of delayed diagnosis of obstetric complications, unavailability of a secure accommodation outside the hospital, reduced compliance of the expectant mother, and absence of guaranteed adequate monitoring at home.^14–16^

Throughout the testing period, patients demonstrated considerable consistency in performing the examinations. The majority of patients adhered to a routine of conducting examinations daily or every other day. This regularity could be seen as a significant indicator of high compliance, especially since this level of consistency was not a requirement for inclusion in the study.

The data from 581 home CTG examinations demonstrated a high success rate (95.7%) in obtaining all necessary diagnostic signals for accurate fetal and maternal health assessment, addressing the existing reservations about the feasibility and reliability of self-administered prenatal monitoring. The primary cause of unsuccessful CTG examinations was the inability to consistently identify uterine contractile activity throughout the test duration. Difficulties in detecting uterine activity are likely linked to the loss of transducer signal during the examination, which could stem from several factors. The probable cause is the improper placement of the tocodynamometer transducer on the abdomen, which is crucial for accurate signal capture.

The measurement of maternal heart rate relies entirely on the pulse oximeter, which captures and integrates it into the recorded data in real-time. The solitary precedent of unsuccessful maternal heart rate detection and evaluation is likely due to either an incorrect placement of the pulse oximeter or a malfunction of the transducer. The rare cases of unrecognized fetal movements might stem from similar technical difficulties, although it is worth noting that in these instances, the women did not report any sensations of fetal movement during the examination. Crucially, not a single examination lacked a fully assessable fetal heart rate detection, along with observed oscillations and oscillation variability. As the devices were programmed to extend the duration of examinations to record a set stretch of uninterrupted fetal heart rate detection, the extensions ensured the successful completion of this component of the detection.

Our results challenge the skepticism associated with transitioning patients with high-risk pregnancies from in-clinic to home care. The data indicate a feasible shift toward more accessible, patient-centered prenatal care, utilizing the advancements in technology to maintain high standards of safety and effectiveness beyond traditional clinical environments.

### Patient acceptance and well-being with home CTG

Home pregnancy care systems have previously been described to enhance patient participation in the medical care process and thus increase patient empowerment, leading to more satisfaction as well as better compliance with the recommendations of medical care workers.^7,17^ This improvement is often attributed to the substantially lower anxiety levels compared with those associated with hospital admission.^5,11^ The findings of this study, presented in Table 1, resonate with these concepts, reflecting similar patterns and observations.

The home CTG examination, while being a medical procedure that can induce anxiety,^18,19^ appears to be less stressful compared with in-clinic tests, with only a small fraction of participants reporting persistent stress connected to examinations throughout the testing period. Crucially, the testing method employed felt secure for the patients who denied feelings of abandonment or insufficient medical supervision. Furthermore, patients reported feeling more comfortable with home CTG examinations compared with those conducted in clinical settings. A frequently mentioned advantage was the time saved by eliminating the need to travel to a medical center. This flexibility encourages patients to conduct tests more frequently than is typically feasible in obstetric clinics. It also enhances inclusivity by facilitating accessible examinations for patients with disabilities or debilitating conditions, such as cancers.

A substantial majority of participants favored home examinations over those conducted in the clinic. Participants universally endorsed their experience with home CTG testing, unanimously recommending it to other patients and expressing a high likelihood of using it in future pregnancies. This demonstrates a high degree of patient satisfaction with the home-based approach, emphasizing that this method not only meets but may even surpass the care and support capabilities of monitoring in conventional clinical settings. Remarkably, only approximately one-third of the participants expressed a desire to conduct all examinations independently, without the necessity for clinic visits. This suggests that the majority of patients view occasional in-clinic testing as an essential part of their obstetric care, which could be attributed to the need for in-person interaction with a medical professional or reservations regarding the novelty of the procedure.

Our significant concern was that patients might feel unable to carry out the examination independently. This apprehension was largely unsubstantiated because most patients rated their proficiency with the procedure as very high and only two needed extra assistance. Although the majority of patients reported no difficulties in performing the examinations, obstacles encountered by the remaining participants should be taken into account. The challenges in operating the systems of the device and accurately positioning the Doppler ultrasound transducer reflect the necessity for certain patients to undergo more comprehensive preparation for independent testing and for the device to offer assistance in the event of improperly conducted examinations. Almost half of the participants reported instances where a CTG examination had to be repeated due to technical issues. In these instances the device is programmed to repeat the examination and does not send the initial, failed attempts to the monitoring center. Consequently, these failed examinations did not affect the overall percentage of interpretable CTGs.

The positive feedback from participants concerning the ease of use, comfort, and flexibility demonstrates a strong patient preference for home-based solutions over conventional regime. Given that accessibility and patient satisfaction are recognized as critical factors in enhancing compliance and adherence to monitoring regimes,^20,21^ the new technology enables more reliable monitoring of high-risk pregnancies. Through this study, we aim to contribute to the ongoing dialogue on incorporating home CTG monitoring into standard prenatal care practices, where the balance between necessary medical oversight and the desire for normalcy and autonomy of patients is delicate.

### Limitations

A limitation of this study is its retrospective approach, which is prone to biases, particularly in the selection of patients. In addition, the small sample size, constrained by the limited number of patients at the clinic who met the eligibility criteria, reduces the chance of observing rare outcomes, which may affect the generalizability of our findings. The additional bias arises from recruitment of study participants from a single clinic, indicating that they may share similar geographic and socioeconomic backgrounds. This could limit the diversity of conditions that are essential for a broad understanding of the applicability of our findings across different populations.

A significant limitation arises from the design of the study, which involves observing a single group of patients examined using the same device model, without employing a control group. Our findings are thus contingent upon the Pregnabit Pro device and patient care system utilized, raising questions about their transferability to different technologies or protocols. The possibility that alternate devices or data collection methods might yield dissimilar outcomes cannot be discounted without further comparative research. In addition, the study does not report and analyze obstetric outcomes of participants, which is a major limitation of this study.

Another limitation stems from the reliance on patient self-reporting for both the home CTG examinations and the accompanying survey. This reliance could negatively impact the consistency of the testing and affect the accuracy with which the survey responses reflect the individual perspectives of the participants. These limitations highlight the need for a cautious interpretation of our findings and suggest areas for improvement in future research efforts.

## Conclusions

Our study indicates that home CTG is interpretable and can be utilized in high-risk pregnancies. It allows for recording a CTG examination that contains all necessary data for accurate fetal health assessment outside of the clinic. The increased comfort and convenience for patients highlight the broader potential of home monitoring to transform the landscape of prenatal care, allowing it to become more flexible and accessible for patients with disabilities and debilitating conditions. Ultimately, our findings advocate for considering the integration of home CTG testing into standard obstetric practice, demonstrating its capability to deliver cost-effective, patient-centered care without compromising quality or outcomes.

## Statement of Originality

This study has not been previously published in any other format or publication.

## Data Availability

The data that support the findings of this study are available from the corresponding author, J.P., upon reasonable request.

## Abbreviations Used

CTG: Cardiotocography
FGR: Fetal Growth Restriction
FIGO: The International Federation of Gynecology and Obstetrics
GDM: Gestational Diabetes Mellitus
NICE: National Institute for Health and Care Excellence
PGDM: Pregestational Diabetes Mellitus
SQUIRE: Standards for Quality Improvement Reporting Excellence
