# Supplementary material for: Home Cardiotocography in High-Risk Pregnancies: A Retrospective Study on Feasibility and Patient Well-Being
Source: Telemed Rep. 2025 Jan 14;6(1):13–22. doi: 10.1089/tmr.2024.0062 (PMC11839534; doi:10.1089/tmr.2024.0062)
Supplement: Supplementary Data S1 [file tmr.2024.0062_supp_datas1.pdf]

Survey for a Patient using the Pregnabit or Pregnabit Pro device

1. Were you able to independently perform the examination after the phone/video training?

- ○ Yes, without any difficulty. I conducted the examination independently.

I needed additional help:

- ○ Consultation with a Midwife.
- ○ Reading the user manual.
- ○ Watching the instructional video on the device.

I couldn't perform the examination independently due to:

- ○ The system being completely non-intuitive.
- ○ Unable to reach the midwife.

2. How do you rate the independent detection of the fetal

Mark the appropriate - where 1 is very difficult and 10 very easy

○ 1 ○ 2 ○ 3 ○ 4 ○ 5 ○ 6 ○ 7 ○ 8 ○ 9 ○ 10

* If it was very difficult – how did you find the heartbeat of the child?

3. Did the CTG examinations proceed without disturbances?

- ○ Without disturbances.
- ○ Examination interrupted. Please state the reason.

4. Have you ever had to repeat a CTG examination for technical reasons?

- ○ No.
- ○ Yes. Please state the reason.

5. Did you feel the need to contact the Midwife during examinations?

- ○ No.
- ○ Yes. In which situations?

6. Which examination was more comfortable for you?

- ○ Conducting the CTG examination independently at home
- ○ CTG examination at a medical center

7. Which statements below best describe your feelings during independent CTG examinations at home?
(check all that apply)

- ☐ I felt comfortable and safe.
- ☐ I felt like nobody was monitoring the examinations.
- ☐ I felt cared for by the midwife and doctor.
- ☐ I felt abandoned.
- ☐ I saved time compared to traveling to a medical facility.
- ☐ Prefer stationary examinations.
- ☐ Felt stressed during independent examinations.
- ☐ Found preparation time-consuming and complicated.
- ☐ Appreciated the opportunity for independent testing at home without hospital/clinic visits.
- ☐ Other, which?

8. What difficulties did you encounter during the monitoring process?

9. Is there anything that would improve your comfort during home CTG examinations?

- No.
- Yes. What?

10. How likely are you to recommend home CTG monitoring to peers or use it in your next pregnancy in the future?

- Very unlikely.
- Rather unlikely.
- Hard to say.
- Rather probable.
- Very probable.

11. Do you think any part of the procedure needs clarification?

- No.
- Yes. What?

12. Do you find conducting independent CTG examinations easy or difficult?

- Easy.
- Difficult.

Thank you for participating in the survey and contributing to the development of our product!

Full name:

Address:

Email:

Phone:

Send
